# Factors related to the prognosis of patients with cerebral aneurysms undergoing microsurgical treatment

**DOI:** 10.1590/1806-9282.20241559

**Published:** 2025-06-02

**Authors:** Adriano Yacubian Yacubian-Fernandes, Leon Cleres Penido Pinheiro, Joao Victor Costa Muller, Pedro Tadao Hamamoto, Marco Antonio Zanini

**Affiliations:** 1 Universidade de São Paulo, Faculty of Dentistry – Bauru (SP), Brazil.; 2 Universidade de São Paulo, Department of Neurology, Faculty of Medicine – Botucatu (SP), Brazil.; 3 Universidade Estadual Paulista Júlio de Mesquita Filho, Faculty of Medicine – Botucatu (SP), Brazil.; 4 Universidade do Estado de São Paulo (São Paulo State University) Campus de Botucatu, Neurologia, Psquiatria e Psicologia, UNESP – Botucatu (SP), Brazil.

**Keywords:** Brain aneurysms, Neurosurgery, Morbidity, Mortality

## Abstract

**BACKGROUND:**

Subarachnoid hemorrhage secondary to a ruptured brain aneurysm should be promptly diagnosed. The angiographic study allows the diagnosis of vascular alterations that determine subarachnoid hemorrhage. The risk of rebleeding is highest in the first 24 h following the first bleeding. The prognosis of the surgical treatment of a brain aneurysm depends on many factors.

**OBJECTIVE:**

The aim of the present retrospective study was to determine which prognostic factors are related to the morbidity and mortality of the microsurgical treatment of different brain aneurysms.

**METHODS:**

A total sample of 371 patients with subarachnoid hemorrhage due to aneurysm bleeding treated by microsurgery from 2013 to 2022 were studied. The variables studied were patient characteristics (age, gender, smoking status, systemic arterial hypertension, and diabetes mellitus), the tomographic findings classified by the Fisher scale, the interval between stroke and surgery, and the clinical condition of patients at admission classified by the Hunt-Hess scale. The primary outcome was the occurrence of death, and the secondary outcomes were the length of hospital stay and the length of stay in the intensive care unit.

**RESULTS:**

The clinical presentation (characterized by the Hunt-Hess scale) at the time of admission of the patient was an important determining factor in the length of hospital stay and intensive care unit stay and the occurrence of death in these patients. Older age and occurrence of diabetes mellitus were also associated with the outcome of death. The surgical moment characterized by the time between stroke and surgery was inversely associated with the outcome of death.

**CONCLUSION:**

The timing for surgery has to be analyzed concerning the clinical presentation of the patient at the time of admission.

## INTRODUCTION

Spontaneous aneurysmal subarachnoid hemorrhage, though rare, leads to significant socioeconomic impact, with higher complications and mortality affecting independence at 6 and 12 months^1^. The predictors of poor outcomes in young adults include aneurysmal size, multiple aneurysms, Hunt and Hess (HH) grade, and hypertension^2–4^. In elderly patients, the predictors include age over 80 years, hypertension, frailty, smoking, high HH grade, multiple aneurysms, and large aneurysmal size^5^. Factors contributing to mortality and morbidity include a high HH grade, intracerebral hematoma, angiographic spasm, rebleeding, and early surgery without vasospasm treatment^6^. Prognostic factors in elderly patients include systemic complications, cerebral infarction, and chronic shunt-dependent hydrocephalus^7^. Significant negative predictors include Fisher 3 bleeding pattern, late cerebral ischemia, early hydrocephalus, and shunt dependence^8^. The presence of a hematoma is a predictor of unfavorable outcomes^9^. Intraoperative aneurysmal rupture does not affect outcomes^10^. In Brazil, the epidemiology of aneurysmal subarachnoid hemorrhage (SAH) is not well-known^11^. Delayed cerebral ischemia is a serious complication^11^. Early surgery for ruptured intracranial aneurysms shows benefits^12^. The timing of surgery varies, with early operation preferred for young, neurologically intact patients^13^. The mortality rate is 35%, with long-term cognitive dysfunction^14^.

## CASUISTRY/METHODOLOGY

### Study design

This retrospective cohort study was conducted to assess the prognostic factors influencing morbidity and mortality in patients with SAH due to cerebral aneurysms who underwent microsurgical treatment at the Hospital das Clínicas de Botucatu – UNESP from January 2013 to December 2022.

A total of 371 patients diagnosed with SAH secondary to aneurysmal ruptures were included in the study. Inclusion criteria consisted of patients aged 18 years and older who underwent microsurgical clipping or coiling of ruptured aneurysms within 14 days of presentation. The case selection was continuous treatment.

The study was approved by the Ethical Committee of the Medical School of Botucatu – São Paulo State University (CAAE-51843021.6.0000.5411/October 2021).

The variables studied were patient characteristics (age, gender, smoking status, systemic arterial hypertension, and diabetes mellitus [DM]), the tomographic picture of the hemorrhage classified by the Fisher scale, the interval between stroke and surgery, and the clinical condition of patients at admission classified by the HH scale.

The primary outcome was the occurrence of death, and the secondary outcomes were the length of hospital stay and the length of stay in the intensive care unit (ICU).

### Statistical analysis

To determine the distribution of the data, the Shapiro-Wilk or Kolmogorov-Smirnov test was used, depending on the sample size. The Mann-Whitney test was used to compare two independent groups with non-parametric data. For variables with a normal distribution, Student's t-test was used to compare the groups. To compare more than two groups, the Kruskal-Wallis test was used, followed by Dunn's test for non-parametric data, and the analysis of variance test followed by the Bonferroni test for parametric data. Spearman's test for non-parametric data and Pearson's test for parametric data were used to study the correlation of variables. The relationship between the independent variables and death was assessed using binary logistic regression. The hazard ratio for this relationship was adjusted for variables that differed between the different groups established by the independent variables. For the outcomes of length of hospitalization and length of ICU stay, a linear regression model was used. Differences were considered statistically significant at p<0.05. The SPSS software (v. 24.0) was used for the analysis.

## RESULTS

The sample consisted of 371 patients who had subarachnoid hemorrhage, of which 287 (77.4%) were females, 84 (22.6%) were males, and 223 (60.1%) were smokers; 259 (69.8%) patients had SAH and 73 (19.7%) had DM; the mean age of the patients was 56.33 (±11.98) years; and the interval between stroke and surgery was 5.46 (±8.45) days. The evaluation at admission indicated patients’ ratings on the HH scale: median 2 (interquartile range [IQR]: 2) and tomography on the Fisher scale: median 3 (IQR: 2).

Regarding the location of the aneurysms, 117 (31.5%) were from the middle cerebral artery (MCA), 109 (29.4%) from the posterior communicating artery (ACoP), 78 (21.0%) from the anterior communicating artery (ACoA), 20 (5.4%) from the pericallosa artery, 11 (3.0%) from the paraclinoid, 10 (2.7%) from the choroidal artery, 10 (2.7%) from the posterior inferior cerebellar artery (PICA), 9 (2.4%) from the carotid bifurcation, 3 (0.8%) from the posterior cerebral artery, 2 (0.5%) from the top of the basilar artery, and 2 (0.5%) from the superior cerebellar artery (SUCA).

The median number of aneurysms was 1, and the median number of surgeries was 1.

The surgical technique can be standardized by a pterional craniotomy in most cases, a microsurgical approach with systematic dissection of the Sylvian cistern and basal cisterns and aneurysmal clipping. Perioperative care was performed in the ICU, and measures to prevent cerebral vasospasm should be highlighted.

The median length of hospital stay was 18.5, and the median length of stay in the ICU was 10.

The number of deaths was 106 (28.6%).

The occurrence of death was associated with DM (p<0.0001) and age (p<0.0001).

Logistic regression for the outcome of death showed that the higher the age, the higher the risk of death. An increase of 1 year of age increased the risk of death by 4.8%. Having DM increased the risk of death by 2.16 times (95%CI 1.151–4.067). A 1-point increase in the HH scale increased the risk of death by 2.5 times. The Fisher grade was not associated with a higher mortality hazard (Table 1).

**Table 1:** Factors related to the risk of death.

Variables	HR	95%CI	p-value
Age	1.048	1.024–1.073	<0.001
Diabetes mellitus	2.164	1.151–4.067	0.017
Hunt–Hess scale score	2.492	1.985–3.128	<0.001

HR: hazard ratio; CI: confidence interval.

The outcome of death was inversely associated with the time of surgery (time between stroke and surgery) (p<0.0001). The earlier the surgery, the higher the occurrence of deaths.

The length of hospital stay was associated with age (p=0.013, r=0.129), hypertension (p=0.025), DM (p=0.043), and HH scale (p<0.001).

According to the linear regression analysis of the outcome of the length of hospital stay, the most important variable was found to be the score on the HH Entry Scale (Table 2).

**Table 2:** Factors related to the length of hospital stay.

Variables	B	95%CI for B	p-value
Age	0.136	-0.011 to 0.283	0.071
Diabetes mellitus	2.187	-2.301 to 6.675	0.339
Systemic arterial hypertension	2.885	-0.964 to 6.734	0.141
Hunt–Hess scale score	2.049	0.680 to 3.418	0.003

CI: confidence interval.

The length of ICU stay was associated with age (p=0.017, r=0.124), HH scale score (p<0.001), and Fisher's scale score (p=0.007).

According to the linear regression analysis of the outcome of the length of intensive care unit stay, the most important variable to predict the length of ICU stay was also found to be the score on the HH scale at admission (Table 3).

**Table 3:** Factors related to the length of intensive care unit stay.

Variables	B	95%CI for B	p-value
Age	0.073	-0.003 to 0.148	0.061
Hunt–Hess scale score	1.757	0.933 to 2.581	<0.001
Fisher's scale score	0.121	-0.948 to 1.190	0.824

CI: confidence interval.

## DISCUSSION

The prognosis of patients who suffered subarachnoid hemorrhage due to aneurysmal rupture and underwent microsurgical treatment may depend on patient characteristics, their bleeding characteristics, and their clinical status after bleeding^15–17^.

In our study, the following patient characteristics were considered: age, gender, smoking status, SAH, and DM. Older age and the occurrence of DM were significantly associated with the outcome of death and the length of hospital stay. The occurrence of hypertension was associated with the length of hospital stay. These associations agree with data from the literature^2,5,7,3^.

The characteristics of hemorrhage were classified by the modified Fisher scale and its severity was not related to the study outcomes. Most of the patients had a computed tomography scan, with hemorrhage classified as Fisher 3.

The mean time between stroke (moment of bleeding) and surgery was 5.46 (±8.45) days and was inversely associated with the occurrence of death. In other words, the earlier the surgery, the higher the occurrence of deaths. It can be considered that the most severe patients (HH scale scores 4 and 5) are operated on earlier, but it can also be considered that early surgery in severe cases worsens the evolution of the patients. The ideal time for microsurgical treatment of brain aneurysms is controversial in the literature^9,12–14^. Early surgery for ruptured intracranial aneurysms is supported as a strategy to improve patient outcomes, reduce the risk of complications, and optimize the management of aneurysmal subarachnoid hemorrhage^18–20^.

Ultra-early surgery for ruptured intracranial aneurysms is generally indicated within the first 24 h post-ictus, and the benefits of this approach are well-documented, especially for good-grade patients^21^.

However, there may be situations where ultra-early surgery may not be indicated. These include cases where there are logistical issues, delayed transfers from other hospitals, late diagnosis, or other nonclinical factors that lead to a delay beyond the 24-h window post-ictus^22^. In such cases, the delay in treatment timing is due to nonclinical reasons rather than clinical decision-making based on the patient's condition. This suggests that the determination of situations where ultra-early surgery is not indicated is more related to external factors impacting the ability to perform surgery within the recommended time frame rather than specific clinical criteria^22,23^.

In older patients, early interventions for surgery may be beneficial to preventing rebleeding and improving outcomes, but a comprehensive evaluation of the patient's condition and risks is essential to determine the optimal timing for surgery^20,24^.

In our series, older patients had lower grades on the HH scale and early surgery was related to mortality, and this is in accordance with the previous explanation that older patients had worse clinical conditions and, in our region, had a delay in reaching our hospital.

Finally, the patient's clinical condition at the time of admission, classified by the HH scale, is the most important indicator for the outcomes of death, length of hospital stay, and length of ICU stay. These findings agree with the literature^2,6,8^.

## CONCLUSION

The clinical presentation (characterized by the HH scale) at the time of admission of the patient with subarachnoid hemorrhage due to aneurysmal bleeding followed by microsurgical treatment is an important determining factor in the length of hospital stay and ICU stay and the occurrence of death in these patients. Older age and occurrence of DM are also associated with the outcome of death. The surgical moment characterized by the time between stroke and surgery is inversely associated with the outcome of death.
